# Memory Window and Endurance Improvement of Hf_0.5_Zr_0.5_O_2_-Based FeFETs with ZrO_2_ Seed Layers Characterized by Fast Voltage Pulse Measurements

**DOI:** 10.1186/s11671-019-3063-2

**Published:** 2019-07-26

**Authors:** Wenwu Xiao, Chen Liu, Yue Peng, Shuaizhi Zheng, Qian Feng, Chunfu Zhang, Jincheng Zhang, Yue Hao, Min Liao, Yichun Zhou

**Affiliations:** 1 Key Laboratory of Low Dimensional Materials and Application Technology of Ministry of Education, School of Materials Science and Engineering, Xiangtan University, Xiangtan, 411105 China; 2 State Key Discipline Laboratory of Wide Band Gap Semiconductor Technology, School of Microelectronics, Xidian University, Xi’an, 710071 China

**Keywords:** HfO_2_-based FeFET, Memory window, Retention, Endurance, ZrO_2_ seed layer

## Abstract

The HfO_2_-based ferroelectric field effect transistor (FeFET) with a metal/ferroelectric/insulator/semiconductor (MFIS) gate stack is currently being considered as a possible candidate for high-density and fast write speed non-volatile memory. Although the retention performance of the HfO_2_-based FeFET with a MFIS gate stack could satisfy the requirements for practical applications, its memory window (MW) and reliability with respect to endurance should be further improved. This work investigates the advantage of employing ZrO_2_ seed layers on the MW, retention, and endurance of the Hf_0.5_Zr_0.5_O_2_ (HZO)-based FeFETs with MFIS gate stacks, by using fast voltage pulse measurements. It is found that the HZO-based FeFET with a ZrO_2_ seed layer shows a larger initial and 10-year extrapolated MW, as well as improved endurance performance compared with the HZO-based FeFET without the ZrO_2_ seed layer. The results indicate that employing of a direct crystalline high-k/Si gate stack would further improve the MW and reliability of the HfO_2_-based FeFETs.

## Background

HfO_2_-based ferroelectric thin films are considered as promising gate-stack materials for ferroelectric field effect transistors (FeFETs) because of their complementary metal-oxide-semiconductor (CMOS) compatibility and scalability. Among several kinds of gate stack structures that can be used in FeFETs, a metal/ferroelectric/insulator/semiconductor (MFIS) represents a more practical configuration because it follows the current MOS device architectures and matches well with the modern high-k metal-gate (HKMG) processes. Therefore, great efforts have been made to design and fabricate FeFETs with MFIS gate stack structures for applications in embedded nonvolatile memories, negative capacitance field effect transistors, artificial neurons, synapses, and logic-in-memory devices [1–8].

Up to now, high-density and fast write speed FeFETs with MFIS gate stack structures have been successfully fabricated using HKMG processes [9, 10]. In addition to the high integration density and fast write speed, a large memory window (MW) and a high reliability with respect to retention and endurance are also critical for employing FeFETs for nonvolatile memory applications [11–14]. Owing to a large band offset to silicon, a high coercive field and a moderate dielectric constant of the HfO_2_-based ferroelectric thin films, HfO_2_-based FeFETs with MFIS gate stack structures exhibit reliable retention properties (10-year extrapolation) [15–17]. However, although the HfO_2_-based thin films demonstrate moderate endurance over 1 × 10^9^ switching cycles [14, 18], HfO_2_-based FeFETs with MFIS gate stack structures have a rather limited endurance ranging from 1 × 10^4^ to 1 × 10^7^ switching cycles [17, 19–23]. Theoretically, employing of high-k insulator layers is expected to reduce the electric field in the MFIS gate stack, which would alleviate the band bending, thereby improving the endurance properties and the MWs of the HfO_2_-based FeFETs [12, 14]. Experimentally, Ali et al. verified that increasing the k value of the ultrathin insulator layer (i.e., using SiON instead of SiO_2_) can effectively improve the endurance properties as well as the MW of the HfO_2_-based FeFETs [13]. In our previous research [24], we reported that the insertion of a crystalline ZrO_2_ high-k layer in the MFIS gate stacks could improve the crystalline quality and suppress the formation of monoclinic phase in Hf_0.5_Zr_0.5_O_2_ (HZO) thin films, which leads to a large MW of 2.8 V characterized by DC voltage sweep method.

In this work, we report on the characterization of the MWs, retention, and endurance of the HZO-based FeFETs with and without crystalline ZrO_2_ seed layers by using fast positive and negative voltage pulse measurements. Moreover, the advantage of employing crystalline ZrO_2_ seed layers on the MW and endurance properties is discussed.

## Methods

**Fig. 1:**
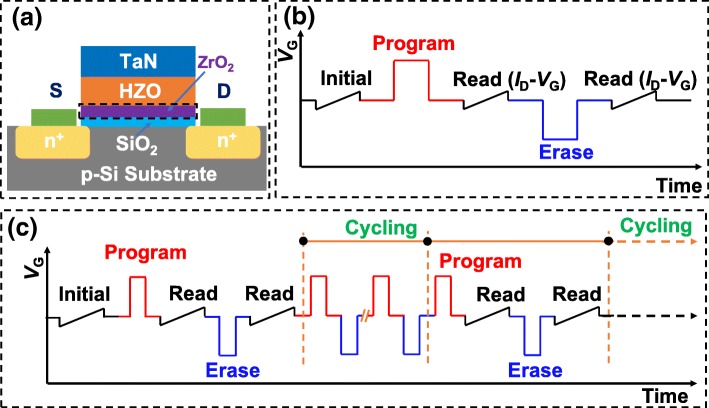
**a** Schematic of the fabricated FeFETs. The additional crystalline ZrO_2_ seed layer is marked by black gridlines. **b**, **c** Test sequences used for MW and endurance measurements

The n-channel FeFETs with and without ZrO_2_ seed layers were fabricated using a gate last process, as described in [24]. The ZrO_2_ seed layer and the HZO layer were both grown at a growth temperature of 300 ^o^C by atomic layer deposition (ALD). The schematic of the fabricated FeFETs is shown in Fig. 1a, whose channel width (*W*) and length (*L*) were 80 and 7 μm, respectively. Meanwhile, TaN/HZO/TaN and TaN/HZO/ZrO_2_/TaN capacitors were also fabricated to evaluate the ferroelectric properties of the HZO thin films. The polarization–voltage (*P–V*) hysteresis loops of the capacitors were measured using a Radiant Technologies RT66A ferroelectric test system, while the device characteristics of FeFETs were measured by an Agilent B1500A semiconductor device analyzer with a pulse generator unit (B1525A) [20]. Two main test sequences used for MW and endurance measurements are shown in Fig. 1b and c. For MW and retention measurements, program/erase (P/E) pulses were first applied to the gates of FeFETs, and read operations were performed at different time intervals using *I*_D_–*V*_G_ sweep (*V*_D_ = 0.1 V) to sense *V*_TH_. Generally, *V*_TH_ is determined as a gate voltage corresponding to a drain current of 10^−7^ A∙W/L [25], and the MW is defined as the difference of *V*_TH_ values between programmed and erased states. For endurance measurements, the MW was measured after a certain number of alternating P/E pulses.

## Results and Discussion

**Fig. 2:**
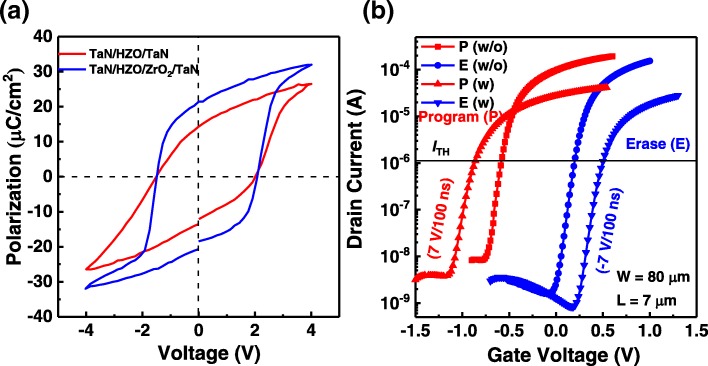
**a** *P–V* hysteresis loops of TaN/HZO/TaN and TaN/HZO/ZrO_2_/TaN MFM structures measured at 4 V and a frequency of 5 kHz. **b** *I*_D_–*V*_G_ curves of HZO-based FeFETs with (w) and without (w/o) ZrO_2_ seed layers after a program pulse (+ 7 V/100 ns) and an erase pulse (− 7 V/100 ns)

Figure 2a shows the *P–V* hysteresis loops of the TaN/HZO/TaN and TaN/HZO/ZrO_2_/TaN capacitors. Remarkably, the TaN/HZO/ZrO_2_/TaN capacitor possesses even better ferroelectric properties than the TaN/HZO/TaN capacitor, which is consistent with the reported results [26], indicating that the crystalline ZrO_2_ seed layer could indeed improve the crystalline quality and suppress the formation of monoclinic phase in HZO thin films [24]. Figure 2b shows the *I*_D_–*V*_G_ curves of the HZO-based FeFETs with and without additional crystalline ZrO_2_ seed layers after P/E pulses. The red symbol lines represent the *I*_D_–*V*_G_ curves after applying a program pulse of 7 V/100 ns, while the blue symbol lines represent the *I*_D_–*V*_G_ curves after applying an erase pulse of − 7 V/100 ns. One can see that the *I*_D_–*V*_G_ curves of both FeFETs show counterclockwise switching characteristics, suggesting that the MWs of the present FeFETs are originated from the polarization switching of the HZO layers, rather than the charge trapping and injection. Nevertheless, the HZO-based FeFET with the additional crystalline ZrO_2_ seed layer exhibits an improved MW of 1.4 V, approximately 1.8 times larger than that (0.8 V) of the HZO-based FeFET without the additional crystalline ZrO_2_ seed layer. Moreover, the obtained MW of 1.4 V is comparable to the best results reported to date [9, 11, 14, 17, 21–23, 27].

**Fig. 3:**
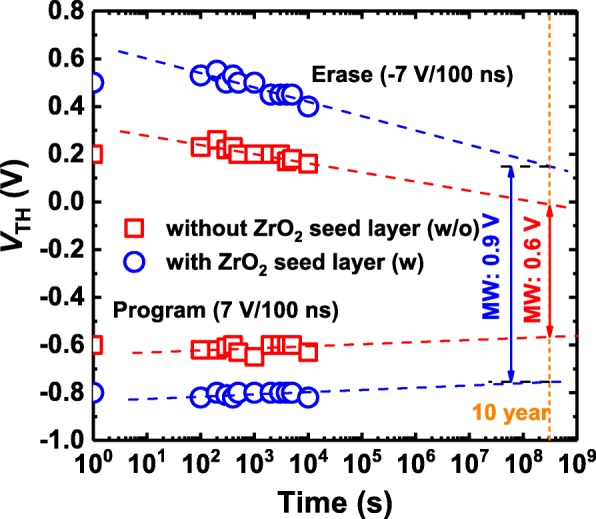
Retention characteristics of HZO-based FeFETs with and without ZrO_2_ seed layers

Reliability with respect to the retention of the HZO-based FeFETs with and without additional crystalline ZrO_2_ seed layers was also evaluated. Figure 3 shows the *V*_TH_ retention characteristics after applying a program pulse of 7  V/100 ns and an erase pulse of – 7 V/100 ns at room temperature. It is clear that the *V*_TH_ values are approximately linear with the logarithmic time scale. The extrapolated MW after 10 years for the HZO-based FeFET with the additional crystalline ZrO_2_ seed layer is 0.9 V, larger than that (0.6 V) for the HZO-based FeFET without the additional crystalline ZrO_2_ seed layer. Since the thick capacitance equivalent thickness (CET) of the ZrO_2_ (1.5 nm)/SiO_2_ (2.6 nm) gate insulator layers would lead to an enhanced depolarization field in the gate stack [13, 15], further improvement in retention properties could be expected if the thickness of the SiO_2_ layer is reduced.

**Fig. 4:**
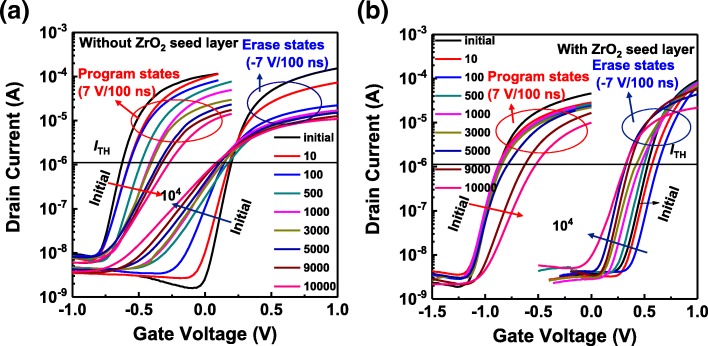
Evolution of *I*_D_–*V*_G_ curves of HZO-based FeFETs **a** without and **b** with ZrO_2_ seed layers with P/E cycling

Figure 4 shows the evolution of *I*_D_–*V*_G_ curves after ± 7 V/100 ns alternating P/E cycles. For the FeFET without the additional crystalline ZrO_2_ seed layer, both significant shift and slope degradation in the *I*_D_–*V*_G_ curves are observed from the early stages of P/E cycling, and the *I*_D_–*V*_G_ curves in the erased states exhibit more slope degradation compared with the program states. For the FeFET with the additional crystalline ZrO_2_ seed layer, although the *I*_D_–*V*_G_ curves in erased states exhibit an obvious positive shift during the early stages of P/E cycling that is attributed to the “wake up” effect [13, 28–32], no obvious shift of *I*_D_–*V*_G_ curves in the program states is observed up to 1 × 10^3^ cycles. Moreover, for the FeFET with the additional crystalline ZrO_2_ seed layer, the *I*_D_–*V*_G_ curves in both erased and program states exhibit only a slight slope degradation up to 1 × 10^3^ cycles.

**Fig. 5:**
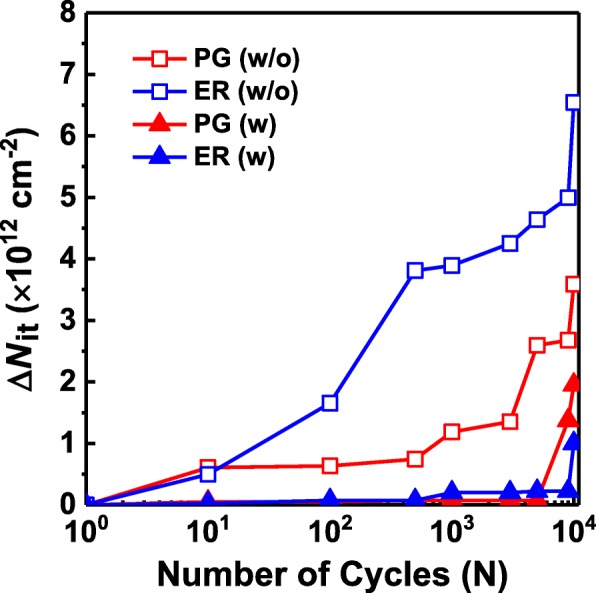
Evolution of ∆*N*_it_ with P/E cycling

According to previous reports [12, 28, 33], the parallel shift in *I*_D_–*V*_G_ curves is attributed to the gradual accumulation of trapped charges in the gate stack, while the slope degradation in *I*_D_–*V*_G_ curves is the result of interface trap generation. Since trapped charges can be de-trapped by electrical means, but generation of interface traps is irreversible, minimizing interface trap generation is extremely important for improving the endurance properties [28]. The interface traps generated by P/E cycling (Δ*N*_it_) can be described using Eq. (1) [34, 35]:

1 $$ \Delta \mathrm{SS}=\frac{\Delta {N}_{it} kT\ln 10}{C_{FI}{\varnothing}_F} $$

where ΔSS is the change of the subthreshold swing, *k* is the Boltzmann constant, *T* is the absolute temperature, *C*_FI_ is the total capacitance of gate stack, and *∅*_*F*_ is the Fermi potential. The Δ*N*_it_ as a function of the P/E cycle for the HZO-based FeFETs with and without additional crystalline ZrO_2_ seed layers is shown in Fig. 5. Clearly, for the FeFET without the additional crystalline ZrO_2_ seed layer, the Δ*N*_it_ increases obviously from the early stages of the P/E cycling, and Δ*N*_it_ in the erased states is much larger than that in the program states. However, the Δ*N*_it_ for the FeFET with the additional crystalline ZrO_2_ seed layer almost does not change up to 1 × 10^3^ cycles, and it is always smaller than that for the FeFET without the additional crystalline ZrO_2_ seed layer. Because inserting the additional ZrO_2_ seed layer reduces the electric field in the gate stack and thus the band bending is weaker, the interface trap generation is alleviated [12, 14].

**Fig. 6:**
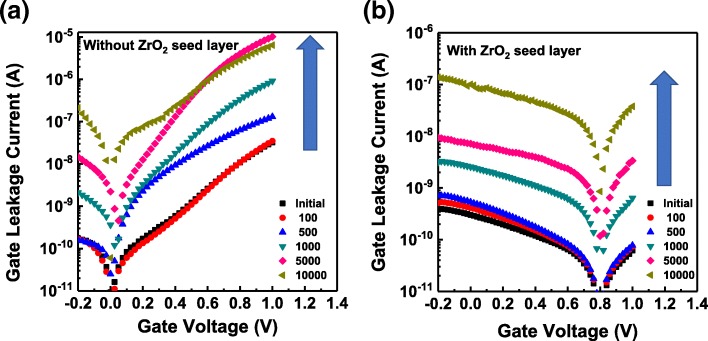
Evolution of gate leakage current characteristics (*I*_G_–*V*_G_ curves) of HZO-based FeFETs **a** without and **b** with ZrO_2_ seed layers with P/E cycling

Figure 6 shows the evolution of gate leakage current characteristics (*I*_G_–*V*_G_ curves) of HZO-based FeFETs with and without ZrO_2_ seed layers with P/E cycling. For the FeFET without the additional crystalline ZrO_2_ seed layer, the gate leakage current increases dramatically from the early stages of the P/E cycling. However, the gate leakage current for the FeFET with the additional crystalline ZrO_2_ seed layer almost does not change up to 5 × 10^2^ cycles, and it is always smaller than that for the FeFET without the additional crystalline ZrO_2_ seed layer. It is reported that the increase in the gate leakage current might be related to the generated interface traps [28]. The reduction in the gate leakage current with cycling for the FeFET with the additional crystalline ZrO_2_ seed layer would be attributed to the suppression of interface trap generation.

**Fig. 7:**
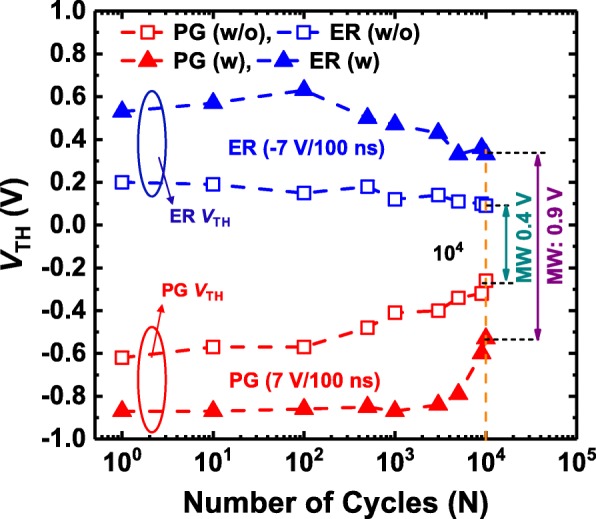
Evolution of *V*_TH_ with P/E cycling

The *V*_TH_ values for program and erase states extracted from the *I*_D_–*V*_G_ curves of the HZO-based FeFETs with and without additional crystalline ZrO_2_ seed layers are shown in Fig. 7. The HZO-based FeFET with the additional crystalline ZrO_2_ seed layer always exhibits a larger MW than the HZO-based FeFET without the additional crystalline ZrO_2_ seed layer. Moreover, the MW of the HZO-based FeFET without the additional crystalline ZrO_2_ seed layer decreases obviously from the early stages of P/E cycling, while the MW of the HZO-based FeFET with the additional crystalline ZrO_2_ seed layer decreases slightly up to 1 × 10^3^ cycles. As the P/E cycling number is further increased, the HZO-based FeFET with the additional crystalline ZrO_2_ seed layer also shows obvious degradation in the slope of the *I*_D_–*V*_G_ curves and the MW, due to the enhanced generation of interface traps. However, the MW of the HZO-based FeFET with the additional crystalline ZrO_2_ seed layer is still larger than 0.9 V up to 1 × 10^4^ cycles, which is approximately 2.3 times larger than that (0.4 V) of the HZO-based FeFET without the additional crystalline ZrO_2_ seed layer. As discussed previously, the decrease of the required electric field for obtaining more saturated polarization states are probably responsible for the improved endurance properties.

## Conclusions

The MWs as well as the reliability with respect to retention and endurance of the HZO-based FeFETs with the TaN/HZO/SiO_2_/Si and TaN/HZO/ZrO_2_/SiO_2_/Si MFIS gate stacks were characterized by fast voltage pulse measurements. The results show that the HZO-based FeFET with the additional crystalline ZrO_2_ seed layer exhibits a large initial memory window of 1.4 V and an extrapolated 10-year retention of 0.9 V, larger than the initial memory window (0.8 V) of the HZO-based FeFET without the additional crystalline ZrO_2_ seed layer. Moreover, the reliability with respect to the endurance of the HZO-based FeFET can be improved by inserting the crystalline ZrO_2_ seed layer in between the HZO layer and the SiO_2_/Si substrate. The MW and endurance improvement of HZO-based FeFETs with ZrO_2_ seed layers are primarily related to the improved crystalline quality of the HZO layer and the suppressed generation of interface traps due to the decrease of the required electric field for obtaining more saturated polarization states. On the basis of this work, it is expected that employing of a direct crystalline high-k/Si gate stack would further improve the MWs and reliability of the HfO_2_-based FeFETs, and thus warrant further study and development.

## Data Availability

The datasets supporting the conclusions of this article are included within the article.

## Abbreviations

CMOS: Complementary metal-oxide-semiconductor
FeFET: Ferroelectric field effect transistor
FeFETs: Ferroelectric field effect transistors
HKMG: High-k metal-gate
HZO: Hf_0.5_Zr_0.5_O_2_
*I*_D_: Drain current
L: Length
MFIS: Metal/ferroelectric/insulator/semiconductor
MW: Memory window
P/E: Program/erase
*P–V*: Polarization–voltage
*SS*: Subthreshold swing
*V*_G_: Gate voltage
*V*_TH_: Threshold voltage
W: Width
Δ*N*_it_: The generated interface traps
